# Isolation of Hepatitis C Virus in Norjizac Vials

**Published:** 2010-03-01

**Authors:** Katayoon Tayeri, Seyed Ramin Radfar, Majid Yaran, Nazila Kassaian, Zary Nokhodian, Behrooz Ataei, Reza Fadaei

**Affiliations:** 1 Isfahan Triangular Clinic, Isfahan University of Medical Sciences, Isfahan, Iran; 2 Infectious Diseases and Tropical Medicine Research Center, Isfahan University of Medical Sciences, Isfahan, Iran

Dear Editor

Hepatitis C virus (HCV) has been recognized as a major health problem worldwide. The estimated number of infected individuals is over 170 million people worldwide [1]. After hepatitis B, it is the most important cause of chronic hepatitis, cirrhosis and hepatocellular carcinoma. Therefore, prevention of HCV infection is one of the most important public health concerns, because the majority of infected people would experience chronic hepatitis [2][3]. HCV can be easily transmitted through blood products transfusion and infected syringes. No surprise, the infection rate is remarkably high among injecting drug abusers (IDUs) [4][5].

HCV has been identified as the most common viral infection affecting IDUs [6]. Sharing contaminated needles and syringes, going to shooting galleries, using cocaine, unprotected sex, and sharing shaving equipments, are all among the major causes of contaminating IDUs with HCV [7][8]. In some countries such as India, Pakistan, Indonesia, and Thailand, the prevalence of anti-HCV antibody among the IDUs is reported to be at least 90% [9]. The reported prevalence from Iran ranged from 38% to 47% [10] and in another study it is about 60% [11].

“Norjizac,” also known as “hand-made Temgizac,” and “Ab Crack (Crack solution),” is a slang name for a drug which is abused by a number of IDUs in Iran since five years ego. It is usually used as intravenous injections, although some of drug abusers use Norjizac intramuscularly and/or subcutaneously. Norjizac is one of the most hard drugs in Iran and accompanying several complications like abscess formation, development of septic emboli and soft tissue infections in IDUs.

Based on reports on probable human contamination in Norjizac vials, we conducted this study to find out why some of IDUs who used the drug have developed HCV infection without any history of sharing in injection tools.

In a case series reported from Isfahan conducted in 2008, 14 vials of Norjizac bought from smugglers from various locations within four months, were tested for HCV. Two-hundred micro-liters from the solution in each vial were obtained and using a high pure viral nucleic acid kit (Roche Diagnostics GmbH, Germany), RNA extraction was done. Thereafter, using Moloney Murine Leukemia Virus (M-MuLV) reverse transcriptase (Fermentas, Life Science), C-DNA was produced. Using specific primers and a probe for the detection of HCV (Tagman probe), PCR was done (Corrbette Research 6000). All laboratory staff was unaware of the nature of the drug under analysis. Overall, two (14%; 95% confidence interval [CI]: 0%–32%) of the 14 tested vials were found positive for HCV with a viral load >104/mL.

We found that Norjizak, itself, can be contaminated with HCV and that avoiding shared needles and syringes is not enough for being infected by HCV in Norjizak abusers. The same thing may be true for other countries that manufacture this drug.

Although conduction of “harm reduction programs,” has resulted in significant decrease in preventing HIV infection among IDUs, the prevalence and incidence of hepatitis C still remains high among this group [12][13][14]. One of the most important points about the survival of HCV is its prolonged stay in the environment. For instance, HCV RNA has been found to be stable in plasma or serum at 40 ºC for seven days [15]. It is believed that HCV can live outside the body for a long time—estimates vary from 7–10 days to weeks or months [16].

Hepatitis C is common among IDUs and needs longterm and expensive treatment courses. In Iran, establishment of the “harm reduction program” has been accepted by the higher governmental authorities and we adapted it to our religious and traditional culture. Harm reduction can work effectively only if it is genuinely supported by an enabling policy and legislative environment [17]. Formerly, we thought that HCV was transmitted only through needle and syringe sharing among the IDUs, but in this study, we found that a new and potentially very dangerous route of viral spread exists.

In Conclusion, in order to decrease HCV infection in IDUs, it is  strongly recommended to follow up drug contamination (especially Norjizac) and develop community awareness about it.
